# α/β-Hydrolase Domain Containing Protein 15 (ABHD15) – an Adipogenic Protein Protecting from Apoptosis

**DOI:** 10.1371/journal.pone.0079134

**Published:** 2013-11-13

**Authors:** Evelyn Walenta, Ariane R. Pessentheiner, Helmut J. Pelzmann, Alexander Deutsch, Madeleine Goeritzer, Dagmar Kratky, Hubert Hackl, Da Young Oh, Andreas Prokesch, Juliane G. Bogner-Strauss

**Affiliations:** 1 Institute for Genomics and Bioinformatics, Graz University of Technology, Graz, Austria; 2 Institute of Biochemistry, Graz University of Technology, Graz, Austria; 3 Division of Hematology, Medical University of Graz, Graz, Austria; 4 Institute of Molecular Biology and Biochemistry, Medical University of Graz, Graz, Austria; 5 Division of Bioinformatics, Biocenter, Innsbruck Medical University, Innsbruck, Austria; 6 Division of Endocrinology and Metabolism, Department of Medicine, University of California San Diego, La Jolla, California, United States of America; Fundação Oswaldo Cruz, Brazil

## Abstract

Our knowledge about adipocyte metabolism and development is steadily growing, yet many players are still undefined. Here, we show that α/β-hydrolase domain containing protein 15 (Abhd15) is a direct and functional target gene of peroxisome proliferator-activated receptor gamma (PPARγ), the master regulator of adipogenesis. In line, Abhd15 is mainly expressed in brown and white adipose tissue and strongly upregulated during adipogenesis in various murine and human cell lines. Stable knockdown of Abhd15 in 3T3-L1 cells evokes a striking differentiation defect, as evidenced by low lipid accumulation and decreased expression of adipocyte marker genes. In preconfluent cells, knockdown of Abhd15 leads to impaired proliferation, which is caused by apoptosis, as we see an increased SubG1 peak, caspase 3/7 activity, and BAX protein expression as well as a reduction in anti-apoptotic BCL-2 protein. Furthermore, apoptosis-inducing amounts of palmitic acid evoke a massive increase of Abhd15 expression, proposing an apoptosis-protecting role for ABHD15. On the other hand, in mature adipocytes physiological (i.e. non-apoptotic) concentrations of palmitic acid down-regulate Abhd15 expression. Accordingly, we found that the expression of Abhd15 in adipose tissue is reduced in physiological situations with high free fatty acid levels, like high-fat diet, fasting, and aging as well as in genetically obese mice. Collectively, our results position ABHD15 as an essential component in the development of adipocytes as well as in apoptosis, thereby connecting two substantial factors in the regulation of adipocyte number and size. Together with its intricate regulation by free fatty acids, ABHD15 might be an intriguing new target in obesity and diabetes research.

## Introduction

During the last decade, obesity became one of the major pandemics and is strongly associated with several diseases, such as type 2 diabetes, liver cirrhosis, cardiovascular diseases, and certain cancers [1,2], leading to socio-economic repercussions [3,4]. Therefore, it is of extreme importance to gain a better insight into adipocyte biology and the link between adipose tissue and disturbed metabolism. 

Obesity is characterized by an excessive increase in number and size of adipocytes, factors tightly regulated by the rate of proliferation of preadipocytes and the differentiation into mature adipocytes [5]. Adipogenesis is a process highly controlled through sequential activation of several genes, most of them transcription factors [6–8]. The nuclear receptor peroxisome proliferator-activated receptor gamma (PPARγ) has been postulated as the master regulator of adipogenesis and is necessary and sufficient for adipocyte differentiation [6,9,10], as many genes of the adipogenesis regulating cascade are either regulated by or regulate PPARγ[11]. Furthermore, in addition to cell division and adipogenesis, it has been demonstrated that apoptosis of pre-adipocytes as well as mature adipocytes is a potent player in the regulation of adipose tissue mass [5]. For instance, adipocyte apoptosis is increased in diet-induced obesity, and inhibition of apoptosis protects from adipose tissue macrophage recruitment, development of fatty liver, and insulin resistance of obese animals [12]. However, the complete mechanism connecting adipogenesis and apoptosis is still elusive. 

We and others utilized high throughput techniques to uncover novel players in adipogenesis [13–16]. Based on previous observations, α/β-hydrolase domain containing protein 15 (ABHD15) was found as being strongly increased during adipocyte differentiation [17]. Previous studies revealed that the insulin-activated protein kinase Akt phosphorylates ABHD15 in adipocytes and that ABHD15 associates with and regulates cyclic nucleotide phosphodiesterase 3B (PDE3B) [17–19]. ABHD15 belongs to the α/β-hydrolase family, which is characterized by a similar tertiary protein fold of α-helixes and β-sheets. However, the family members do not share obvious sequence similarities, leading to a widespread variety of enzyme subclasses, such as lipases, esterases, dehydrogenases, dehalogenases, peroxidases, and epoxide hydrolases [20]. It is therefore expected that ABHD15 possesses a hydrolytic active site but its distinct function has not been defined so far.

In this study, we demonstrate that Abhd15 is required for adipogenesis and a direct and functional target gene of PPARγ, resulting in strongly increased Abhd15 expression during murine and human adipogenesis. Additionally, we identified free fatty acids (FFAs) as negative regulators of Abhd15 expression in differentiated adipocytes as well as in physiological circumstances like in fasting or obesity. Finally, we show that Abhd15 knockdown results in increased apoptosis, whereas induction of apoptosis increases Abhd15 expression, suggesting a protective role of ABHD15 against apoptosis.

## Materials and Methods

### Animal studies

All animal procedures followed the National Institute of Health Guidelines for the Care and Use of Laboratory Animals and were approved by the Austrian Ministry for Science and Research. Male C57BL/6 (age mentioned in figures and text) and 4 months old male ob/ob mice were used for this study. Animals were kept on a 12/12 hours light/dark cycle and were put on either chow or high fat diet (60% calories in fat; Ssniff, Soest, Germany) with 8 weeks of age. Tissues were harvested from mice in *fed ad libitum* state or after fasting for 12 hours. 

### Promoter analyses

Genome organization around the Abhd15 transcription start site was visualized using the UCSC genome browser (GRCm38/mm10). Custom tracks include data from chromatin immunoprecipitation (ChIP) followed by sequencing or microarray analysis, respectively, for PPARγ at day 6 [21] and for PPARγ and C/EBPα at day 10 [22] during 3T3-L1 adipocyte differentiation, as well as for PPARγ-RXRα direct repeats 1 (DR1) motifs (similarity score > 0.90) (potential binding sites on the plus strand are shown in red and on the minus strand in blue). *In silico* promoter analysis was performed with a Perl implementation of the MatInspector algorithm [23] using a 1133 element position weight matrix (PWM) as identified before [22]. Sequence logo was generated using http://icbi.at/logo.

### Cell culture, adipocyte differentiation, and lipid staining

Cells were cultured as described before [16]. 3T3-L1 adipocytes were treated with 1 µM rosiglitazone at time points and durations indicated in the text, figures and figure legends. Fully differentiated cells (day 7 after differentiation start) were treated with 0.5 mM 3-isobutyl-1-methylxanthine, 10 µM isoproterenol, or 100 µM palmitic acid in serum-free high glucose DMEM containing L-glutamine (2 mM), penicillin (50 U/mL) and streptomycin (50 µg/mL) (P/S), and harvested after 2 hours of treatment. Preconfluent cells were treated with palmitic acid concentrations as indicated in the text, figures, and figure legends for 24 hours. Palmitic acid was resolved in 90% ethanol to a stock of 50 mM and added to serum-free high glucose DMEM containing L-glutamine, P/S, and 0.5% BSA. Plates were oil red O-stained as described earlier [24]. MEFS [25,26], OP-9 [16] and SGBS [16] cells were cultured as described before.

### RNA isolation, reverse transcription, and gene expression analysis

Cells were washed with PBS and harvested using an RNA isolation kit (Marcherey-Nagel, Dueren, Germany). Tissue RNA was isolated with the TRIzol® reagent (Invitrogen, Carlsbad, USA) according to the manufacturer’s protocol. Expression of genes was assessed by real-time reverse transcriptase-polymerase chain reaction (RT-PCR) using an ABI Prism 7700 Sequence Detector system utilizing SYBR Green PCR master mix (Applied Biosystems, Darmstadt, Germany). Gene expression was normalized using TFIIβ for murine tissues and cells and β-actin for human cells as reference genes. Relative mRNA expression levels were calculated using averaged 2^-ddCt^ values for each biological replicate as implemented before [27]. 

Primer sequences: 

mAbhd15 (TATGAACGTGGGTTCTTGCT, TTGGTGTGACAGAACAGGGT), 

hAbhd15 (CCGTGCTGCGCTGCCGAGAGTGG, GGCTGTGGCATACCTGCTGAGGGCG), 

hβ-Actin (CGCCGCATCCTCCTCTTC, GACACCGGAACCGCTCATT), 

mC/ebpα (ATCTGCGAGCACGAGACGTC, TGTCGGCTGTGCTGGAAGA), 

mFabp4 (CGACAGGAAGGTGAAGAGCATC, ACCACCAGCTTGTCACCATCTC), 

mFasn (GCTGTAGCACACATCCTAGGCA, TCGTGTTCTCGTTCCAGGATC), 

mPparγ2 (TGCCTATGAGCACTTCACAAGAAAT, CGAAGTTGGTGGGCCAGAA), 

mTFIIβ (GTCACATGTCCGAATCATCCA, TCAATAACTCGGTCCCCTACAA)

### Silencing of Abhd15 using short hairpin (sh)RNA lentivirus particles

One control non-targeting shRNA lentivirus and two shRNA lentiviruses directed against Abhd15 were purchased from Sigma (MISSION shRNA lentiviral particles NM_026185). 3T3-L1 cells were seeded into 6-well plates 12 hours before transduction using 3*10^4^ cells/well (30% confluence). Cells were infected over night with 5 MOI (multiplicity of infection) in standard medium containing 8 µg/ml polybrene (Sigma). After 16 hours, the infection medium was replaced with fresh medium containing 3 µg/mL puromycin (Sigma). 3T3-L1 cells were selected for stable expression for at least 5 days. 

### Silencing of Abhd15 via electroporation using siRNA

Control non-targeting siRNA and siRNA directed against Abhd15 were purchased from Sigma (MISSION siRNA NM_026185). 80,000 fully differentiated 3T3-L1 (day 8 after differentiation start) were electroporated per 10 µL reaction with siRNA (100 nM) using the Neon Transfection System (Invitrogen, Carlsbad, USA), at 1400 V, 20 ms, 1 pulse. Cells were harvested 2 days after transfection.

### Generation of recombinant retrovirus

The coding sequence of mouse Abhd15 was amplified by PCR from mouse adipose tissue cDNA using *Pfu* polymerase (Thermo Scientific, Waltham, USA). The primers were designed to create *Bgl*II and *Xho*I restriction sites and the product, containing the whole open reading frame, was ligated into *Bgl*II-*Xho*I digested Murine Stem Cell Virus vector (pMSCV puro; BD Biosciences Clontech). To produce infectious, but replication-incompetent recombinant retroviruses expressing Abhd15, PhoenixEco packaging cells were transfected with pMSCV-Abhd15 using Metafectene (Biontex Laboratories, Planegg, Germany). Supernatants containing viral particles were collected 48 hours after transfection. Viral supernatants were supplemented with 8 µg/mL polybrene and added to 3T3-L1 cells (30% confluence) for infections for 18–24 hours. Cells were selected with 3 µg/mL puromycin, expanded, and seeded for differentiation experiments. The empty pMSCVpuro vector was used as control.

### Western blot analysis

Control (ntc) and Abhd15-silenced (Abhd15_sil1) 3T3-L1 cells were harvested by scraping with lysis buffer (50 mM Tris-HCl pH 6.8, 10% glycerol, 2.5% SDS, 1x protease inhibitor cocktail, 1 mM PMSF) after two washing steps with PBS and benzoase (Merck, Vienna, Austria) digested. Protein concentration was determined with the BCA protein assay kit (Pierce, Rockford, USA). Protein samples were separated according to size by SDS-polyacrylamide gel electrophoresis (NuPAGE, Invitrogen). Resolved samples were transferred onto nitrocellulose or polyvinylidene difluoride membranes. Blots were incubated with an anti-rabbit polyclonal antibody against ABHD15 (1:1 kind gift from Gustav Lienhard), against a monoclonal anti-mouse β-actin antibody (1:25,000 Sigma), or anti-rabbit polyclonal antibodies BCL-2 (1:1000), and BAX (1:1000) (Cell Signaling Technology, Danvers, MA), or against a monoclonal anti-mouse β-ACTIN antibody (1:20,000 Santa Cruz, Heidelberg, Germany). The horseradish peroxidase-conjugated goat anti-mouse (1:3000 for ABHD15 antibody, 1:2000 for BCL-2 and BAX antibodies) and rabbit anti-mouse (1:3000 for the β-ACTIN antibody from Sigma, 1:1000 for the β-ACTIN antibody from Cell Signaling) antibodies (Dako, Glostrup, Denmark) were visualized by enhanced chemiluminescence detection (ECL component from Pierce Clarity^TM^ and Western ECL Substrate from Bio-Rad, Hercules, USA) using a ChemiDoc^TM^ MP Imaging System (Bio-Rad). 

### Luciferase reporter assays

Three regions upstream of the Abhd15 transcription start site (TSS) (F1 -1190-0bp, F2 -1190-530bp, and F3 -530-0bp from TSS) were cloned into luciferase reporter vectors (Promega, Madison, USA) either containing a minimal promoter (F2 into pGl4.26) or not (F1 and F3 into pGL4.21), and were cotransfected with Pparγ2 and Rxrα containing pCMX expression vectors. As described before [28], renilla reporter vector pGl4.75 (Promega, Madison, USA) was cotransfected in all experiments in a ratio of 1:50 to luciferase reporter vectors as a control for varying transfection efficiencies. Transfection into Cos7 cells was performed in 96-well plates using MetafectenePro (Biontex, Martinsired, Austria) according to the manufacturer’s protocol in a ratio of MetafectenePro to DNA 3:1 (µl:µg). 100 ng of luciferase reporter vector and either 50 ng of Pparγ2 and Rxrα or 100 ng of the empty pCMX as a control were used. After 48 hours cells were lysed and assayed according to the protocol provided with the Dual-luciferase assay system (Promega, Madison, USA). Luminescence read-outs were generated with a Berthold Orion II luminometer. Relative luciferase activity was calculated by referring renilla-normalized values to empty luciferase vector measurements. 

### Assessment of cell growth

Cells were plated at a density of 1000 cells/96-well and cultured for 72 hours. Seven replicates of the CellTiter 96 AQueous One Solution Cell Proliferation Assay (Promega, Madison, USA) were measured using 3-(4,5-dimethylthiazol-2-yl)-5-(3-carboxymethoxyphenyl)-2-(4-sulfophenyl)-2*H*-tetrazolium, inner salt (MTS). Absorbance was recorded by a BioRad spectrophotometer at 490 nm.

### BrdU cell cycle analysis

1x10^6^ cells were incubated for 1 hour at 37°C with 10 µM BrdU solution. BrdU and 7-AAD staining was performed according to the BrdU Flow kit manual (Becton Dickinson, San Diego, USA). A total of 1×10^5^ events were collected on FACScan and cellular DNA content was analyzed by FlowJo software (TreeStar, Ashland, USA).

### Caspase-Glo 3/7 assay

14,500 cells/96-well (in 100 µL) were cultured for 18 hours and analyzed for caspase activation using the Caspase-Glo 3/7 assay (Promega Corporation, Madison, USA), according to the manufacturer's protocol. Luminescence was measured 30 min after adding the Caspase-Glo 3/7 reagent (Caspase-Glo substrate and buffer).

### Statistical analysis

If not otherwise stated, results are mean values (±standard deviation) of at least three independent experiments. Statistical significance was determined using the two-tailed Student’s *t* test.

## Results

### Abhd15 is a direct and functional target gene of PPARγ

**Figure 1:**
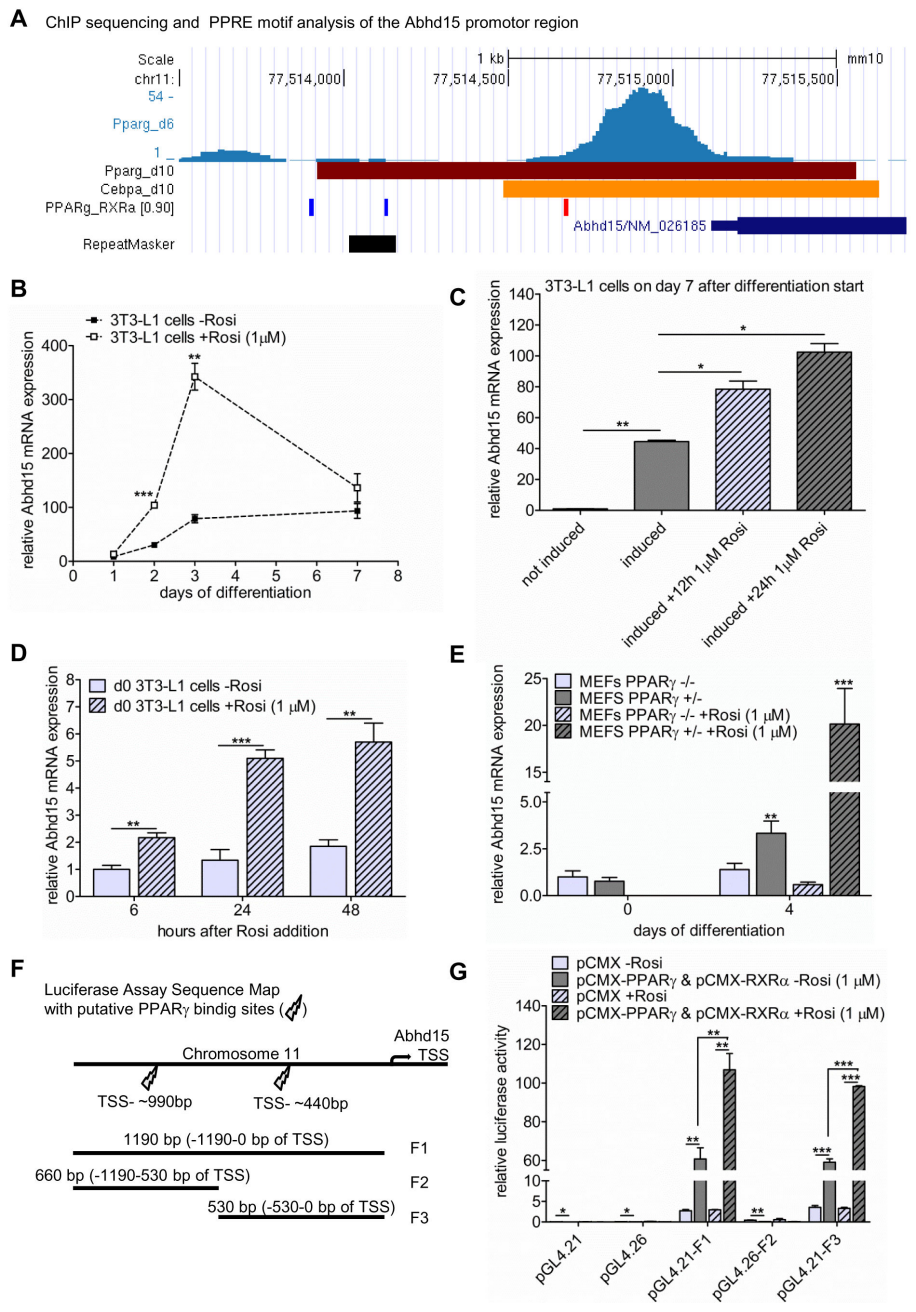
Abhd15 is a direct and functional PPARγ target gene. **A**. Genome organization around the Abhd15 transcription start side (TSS) of 3T3-L1 cells during differentiation with ChIP data of peroxisome proliferator-activated receptor gamma (PPARγ) (day 6 and day 10) and CCAAT-enhancer-binding protein alpha (C/EBPα) (day 10) binding, and Pparγ-Retinoid X receptor (RXRα) direct repeat motif analysis. The data suggest putative PPARγ-RXRα binding ~990 bp and ~440 bp upstream of the Abhd15 TSS. **B**-**D**. Abhd15 mRNA levels of 3T3-L1 cells upon PPARγ agonist rosiglitazone (Rosi) treatments. Cells were treated with 1 µM Rosi (**B**) during differentiation, (**C**) for 12 and 24 hours on day 7 of differentiation, and (**D**) for 6, 12, and 24 hours before induction of differentiation, all leading to increased Abhd15 expression. **E**. Abhd15 mRNA expression in Pparγ -/- and Pparγ +/- mouse embryonic fibroblasts (MEFs). Abhd15 is hardly expressed in Pparγ -/- MEFs and can only be further increased upon addition of Rosi (1 µM) in Pparγ +/- MEFs. **F**. Sequence map of the sequences containing either one (F2 and F3) or two (F1) of the putative PPARγ-RXRα binding sites, evaluated in figure A, used for the luciferase assay. **G**. The 3 regions of interest located upstream of the Abhd15 gene were cloned into luciferase reporter vectors (named pGL4.21-F1, pGL4.26-F2, pGL4.21-F3) and cotransfected with either Pparγ/Rxrα expressing vectors or an empty vector (pCMX) into Cos7 cells. The luciferase activity of pGL4.21-F1 and pGL4.21-F3, both containing the putative PPARγ-RXRα binding site ~440 bp upstream to the TSS, were significantly increased when compared to pCMX-transfected cells. Addition of Rosi to cells cotransfected with pGL4.21-F1 or pGL44.21-F3 and Pparγ/Rxrα, again significantly increased luciferase activity. Data is presented as mean ± SD from at least three independent experiments. Statistical significance was determined using the two-tailed Student’s t-test. *p<0.05, **p<0.01, ***p<0.001.

In a search for new key players of adipogenesis, we surveyed published ChIP sequencing data sets that identified genome-wide PPARγ and CCAAT-enhancer-binding protein alpha (C/EBPα) binding sites in differentiating 3T3-L1 cells [21–23]. In these studies, Abhd15 possesses PPARγ and C/EBPα binding sites in its promoter region (Figure 1A). Further, motif search for peroxisome proliferator response element sequences (PPRE) revealed two putative binding sites of PPARγ and its dimerization partner retinoid X receptor alpha (RXRα), ~990 bp and ~440 bp upstream to the Abhd15 transcription start site (TSS) (Figure 1A). Together with the upregulation of Abhd15 during differentiation of 3T3-L1 cells (Figure 1B), these findings suggest that Abhd15 might be regulated by PPARγ. In order to test this hypothesis, 3T3-L1 cells were exposed to the PPARγ agonist rosiglitazone (1 µM). As expected, the treatment during differentiation led to strongly increased mRNA expression of Abhd15 (Figure 1B). Furthermore, short term treatments of fully differentiated 3T3-L1 adipocytes with rosiglitazone for either 12 or 24 hours (Figure 1C), and undifferentiated cells for 6, 12, or 24 hours (Figure 1D) showed a time-dependent increased mRNA expression of Abhd15. Additionally, mouse embryonic fibroblasts (MEFs) isolated from Pparγ -/- and Pparγ +/- mice [26] were subjected to hormone-induced adipocyte differentiation. While Pparγ +/- MEFs showed significantly increased Abhd15 mRNA levels from day 0 to day 4 of differentiation, Pparγ -/- MEFs did not (Figure 1E). Moreover, the addition of rosiglitazone to Pparγ +/- MEFs increased Abhd15 expression 6-fold on day 4, whereas in Pparγ -/- MEFs rosiglitazone did not evoke any changes in expression level (Figure 1E). Finally, in order to prove the direct binding of PPARγ and its dimerization partner RXRα to the Abhd15 promoter region, luciferase reporter assays with three different sequences were performed (segments containing the 990 bp PPRE (F2), the 440 bp PPRE (F3), and one segment containing both (F1) (Figure 1F). We clearly observed Abhd15 promoter activation of the region ~440 bp upstream to the TSS, which could be further increased upon addition of rosiglitazone (Figure 1G). The region with the putative PPRE at ~990 bp seemed not to be involved in Abhd15 promoter activation (Figure 1G).

Taken together, these results indicate that Pparγ is a prerequisite for Abhd15 expression and that Abhd15 is a direct and functional PPARγ target gene.

### Abhd15 expression is upregulated during adipogenesis and decreased by high FFA levels

**Figure 2:**
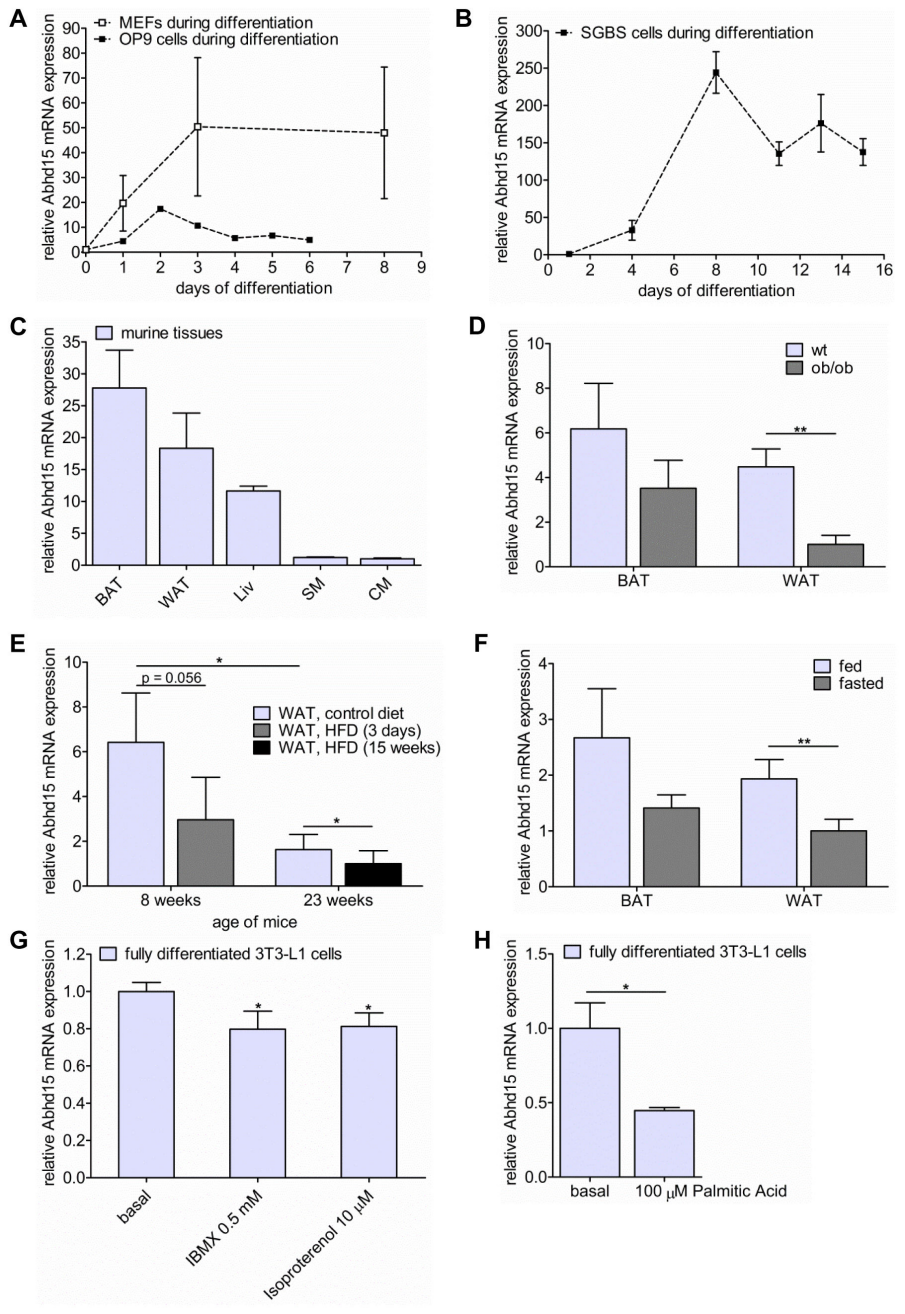
Abhd15 expression is regulated during adipogenesis and decreased by elevated free fatty acid levels. **A**-**B**. Abhd15 mRNA expression is increased during adipocyte differentiation of (**A**) OP 9 cells, mouse embryonic fibroblasts (MEFs), and (**B**) human Simpson-Golabi-Behmel syndrome (SGBS) cells. **C**. Abhd15 mRNA is highly expressed in brown and white adipose tissue (BAT and WAT), to a lower extent in liver (Liv), and hardly in skeletal (SM) and cardiac muscle (CM) of wild-type mice in the fed state. **D**. Abhd15 mRNA expression is decreased in WAT and BAT of genetically obese mice (ob/ob) compared to wild type (wt) mice. **E**. Mice fed a high fat diet (HFD, 60% calories in fat) show a decreased Abhd15 mRNA expression in WAT already after 3 days, but still after 15 weeks on this diet. Additionally, aging strongly decreases Abhd15 mRNA levels. **F**. Abhd15 mRNA expression is regulated depending on the nutritional status in mouse tissues. Upon fasting, the expression is decreased in both BAT and WAT. **G**. Simulated fasting of fully differentiated 3T3-L1 cells (day 7 of differentiation) with IBMX (0.5 mM) and isoproterenol (10 µM) for 2 hours resulted in reduced Abhd15 mRNA expression. **H**. Treatment of fully differentiated 3T3-L1 cells (day 7 of differentiation) with palmitic acid (100 µM) strongly reduces Abhd15 mRNA expression. Data is presented as mean ± SD from at least three independent experiments. Statistical significance was determined using the two-tailed Student’s t-test. *p<0.05, **p<0.01.

Next, gene expression of Abhd15 was assessed in human and murine model systems of adipogenesis. In addition to its upregulation in 3T3-L1 cells (Figure 1B), Abhd15 was strongly upregulated during adipogenic differentiation of OP 9 cells and MEFs (Figure 2A). A similar expression profile of the human ortholog of Abhd15 could be shown in Simpson-Golabi-Behmel syndrome (SGBS) cells (Figure 2B). In accordance to the increased expression during adipogenic differentiation, Abhd15 was mainly expressed in murine brown (BAT) and white adipose tissue (WAT), to a lower extent in liver, and hardly in skeletal (SM) and cardiac muscle (CM) (Figure 2C). Interestingly, Abhd15 mRNA expression was significantly decreased in WAT of genetically obese, leptin-deficient mice (ob/ob) compared to their wild type littermates (Figure 2D). Moreover, already after 3 days on a high fat diet (HFD), Abhd15 mRNA expression was strongly down-regulated in WAT when compared to chow-fed controls (Figure 2E). This reduction of Abhd15 mRNA expression in WAT was still evident after 15 weeks on HFD (Figure 2E). Notably, 23 weeks old mice had strongly reduced expression levels compared to 8 weeks old littermates, suggesting that Abhd15 mRNA expression is reduced in an age-dependent manner (Figure 2E). Furthermore, overnight fasting decreased Abhd15 mRNA expression levels in murine WAT and BAT (Figure 2F). Simulated fasting in mature adipocytes by short-term treatment (2 hours) of fully differentiated 3T3-L1 cells with isoproterenol or 3-isobutyl-1-methylxanthine (IBMX) also resulted in reduced Abhd15 mRNA expression (Figure 2G). Both components increase intracellular cAMP levels and thereby stimulate lipolysis [29,30].

FFA levels are increased in diet- [31] and genetically-induced [32] obesity, fasting [33] and aging [34]. Therefore, the observations that Abhd15 mRNA expression is reduced in obese mice, in mice fed HFD, but also upon fasting indicate that increased FFAs, the common denominator in these conditions, directly diminish Abhd15 expression. In accordance, short-term treatment (2 hours) of mature adipocytes with 100 µM palmitic acid, a dose reflecting fasting levels without evoking toxic effects [35], strongly reduced Abhd15 mRNA expression (Figure 2H).

### Abhd15 is required for adipogenesis

**Figure 3:**
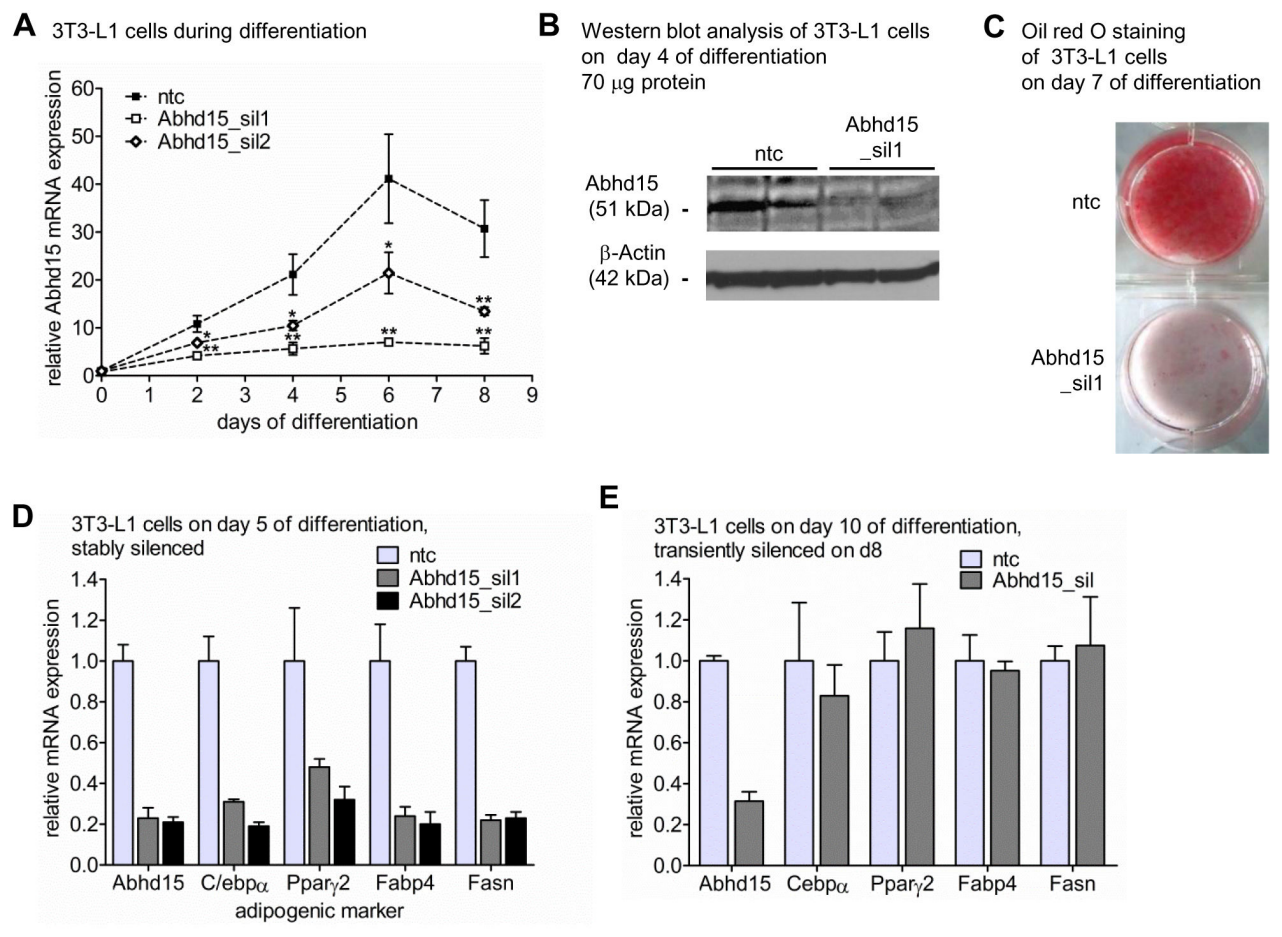
Abhd15 expression is required for adipogenesis. **A**-**D**. 3T3-L1 cells were infected with lentiviral particles coding for Abhd15 shRNA (Abhd15_sil) or using a non-target shRNA as control (ntc), selected for puromycin resistance, expanded as a mixed population and differentiated. **A**. Silencing efficiency during adipogenesis of two knock-down lentiviruses against Abhd15, determined by qPCR assay. **B**. Protein was harvested at day 4 of differentiation of control (ntc) and Abhd15-silenced 3T3-L1 cells (Abhd15_sil1) and subjected to western blotting using the anti-Abhd15 antibody. β-actin served as loading control. Abhd15 protein expression is decreased in Abhd15-silenced 3T3-L1 cells compared to control cells. n=2 C. Silencing of Abhd15 impairs adipogenesis, indicated by the strongly decreased amount of neutral lipids on day 7 of differentiation, stained with Oil red O. **D**. Stable silencing of Abhd15 in 3T3-L1 cells showed high influences on the expression levels of various important adipogenic genes on day 5 of differentiation (Cebpα, Pparγ, fatty acid binding protein 4 (Fabp4), fatty acid synthase (Fasn)). **E**. Transient silencing of Abhd15 by electroporation of siRNAs on day 8 of differentiation did not show any effects onto the mRNA levels of adipogenic genes in fully differentiated 3T3-L1 cells (day 10). Data is presented as mean ± SD from at least three independent experiments if not otherwise stated. Statistical significance was determined using the two-tailed Student’s t-test. *p<0.05, **p<0.01, ***p<0.001.

To gain more insight into its function, stable knock-down of Abhd15 in 3T3-L1 cells was performed. For this purpose, an shRNA construct targeting Abhd15, encoded by lentiviral vectors, was used to generate 3T3-L1 cells with constitutive knock-down of Abhd15 expression. After transduction and selection, the cells were grown to confluence and induced to differentiate using a standard hormonal cocktail. During differentiation, two Abhd15-targeting shRNA lentiviral constructs revealed a reduction of up to 80% compared to a non-targeting control shRNA (ntc) on mRNA level (Figure 3A). The silencing of the stronger shRNA lentiviral construct (Abhd15_sil1) was confirmed on protein level using western blot analysis (Figure 3B). Knock-down of Abhd15 drastically reduced the formation of lipid droplets, as revealed by oil red O staining of fully differentiated 3T3-L1 cells (Figure 3C). Additionally, mRNA expression levels of several adipogenic markers, such as C/ebpα, Pparγ, fatty acid binding protein 4 (Fabp4), and fatty acid synthase (Fasn), were decreased in Abhd15-silenced compared to control cells at day 5 of differentiation (Figure 3D). However, stable overexpression of Abhd15 (Panel 1 in Figure S1) did not induce any changes in the differentiation capacity of 3T3-L1 cells (Panel 2 in Figure S1).

In order to investigate a potential influence of Abhd15 on mature adipocytes, Abhd15 was transiently knocked down in fully differentiated 3T3-L1 cells by means of siRNA introduced by electroporation. Although the expression level of Abhd15 was reduced by 70% in mature adipocytes (Figure 3E), neither differences in lipid accumulation (data not shown), nor changes in expression levels of C/ebpα, Pparγ, Fabp4, and Fasn could be detected (Figure 3E). 

Together, these results point out that Abhd15 is a required factor for adipogenic differentiation, whereas reduced Abhd15 expression in mature adipocytes has no effect on the maintenance of the differentiated status. 

### Abhd15 expression is tightly connected to apoptosis

**Figure 4:**
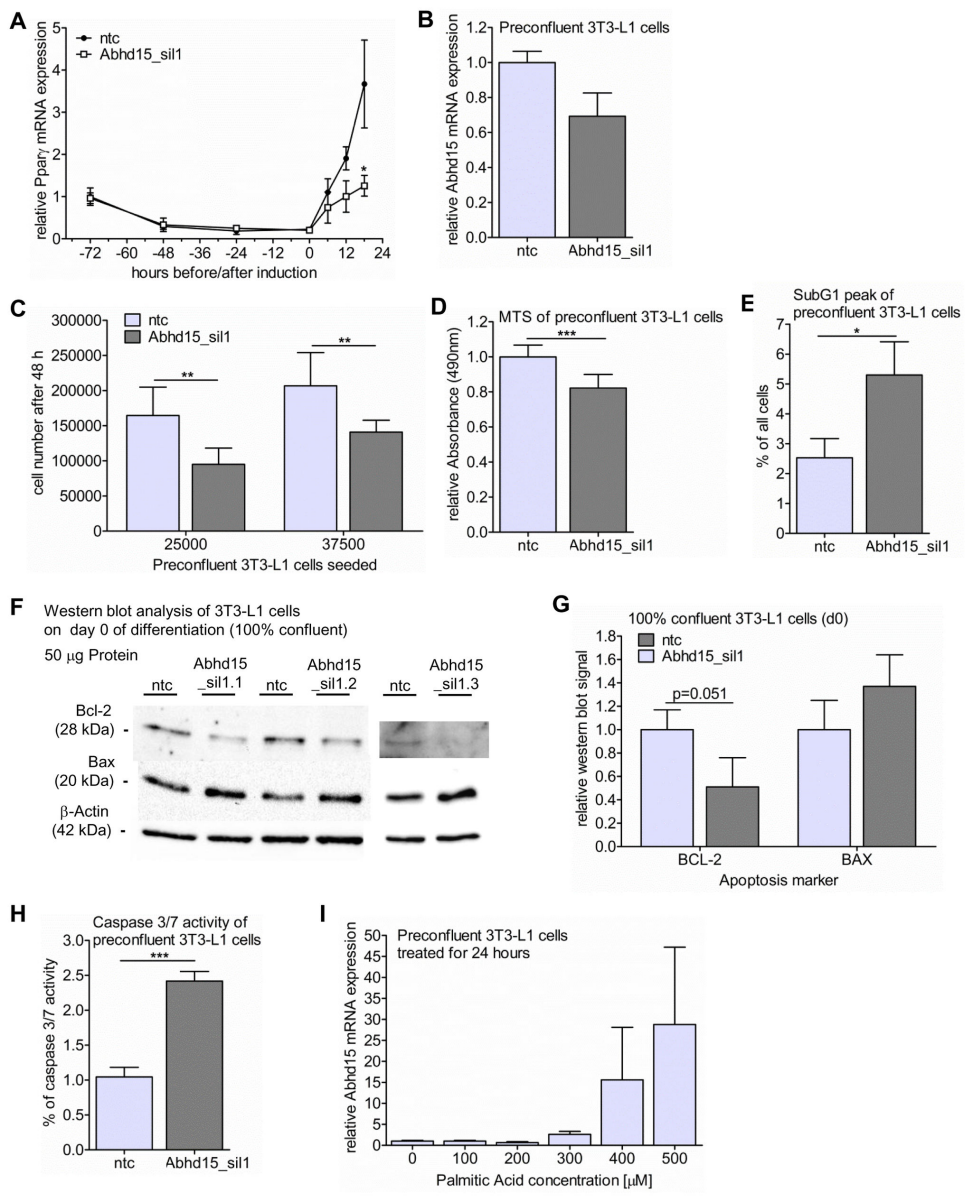
Abhd15 expression is tightly connected to apoptosis. **A**-**H**. 3T3-L1 cells were infected with lentiviral particles coding for Abhd15 shRNA (Abhd15_sil) using a non-target shRNA as control (ntc), selected for puromycin resistance, and expanded as a mixed population. **A**. After inducing 3T3-L1 cells to differentiate, Pparγ mRNA expression did not increase to the same extent in Abhd15-silenced cells as in control cells. **B**. Silencing efficiency of Abhd15 on mRNA level in preconfluent cells reached ~30%. **C**. Cell proliferation is reduced in Abhd15-silenced preconfluent 3T3-L1 cells, shown by the decreased cell number compared to control cells 48 hours after seeding. **D**. The colorimetric proliferation assay (MTS) showed a reduction in proliferation of preconfluent Abhd15-silenced cells by ~20%. **E**. Analysis of preconfluent 3T3-L1 cells, using BrdU FACScan, showed a strongly elevated SubG1 peak, pointing towards increased apoptosis. **F**-**G**. Western blot (**F**) and relative western blot signals (**G**) of the essential regulators of apoptosis B-cell lymphoma 2 (BCL-2) and BCL-2-associated X protein (BAX). The protein expression of the pro-survival regulator BCL-2 was decreased, while the protein level of the pro-apoptotic regulator BAX increased. **H**. Increased caspase 3/7 activity could be measured in preconfluent Abhd15-silenced 3T3-L1 cells, proofing increased apoptosis. **I**. 24 hours treatment of preconfluent 3T3-L1 cells with palmitic acid concentrations, reaching from non-apoptotic (100 µM) to apoptosis-inducing (500 µM) [45], increased Abhd15 mRNA expression dose dependently. Data is presented as mean ± SD from at least three independent experiments. Statistical significance was determined using the two-tailed Student’s t-test. *p<0.05, **p<0.01, ***p<0.001.

To track the origin of the differentiation defect in Abhd15-silenced 3T3-L1 cells, we closely monitored the mRNA expression of Pparγ during early differentiation. Right after induction the expected increase in Pparγ expression was reduced in Abhd15-silenced cells compared to control cells (Figure 4A), hinting at an early defect of differentiation. In 3T3-L1 cells, the first steps before terminal differentiation include growth arrest due to cell-cell contact, followed by two sequential rounds of mitosis (called mitotic clonal expansion), which are necessary for terminal differentiation [36]. Mitotic clonal expansion involves a transcription factor cascade, followed by the expression of genes responsible for the adipocyte phenotype [37]. The reduced Pparγ levels upon Abhd15 silencing started right during this phase of mitotic clonal expansion, suggesting a cell cycle defect due to reduced Abhd15 expression. Preconfluent Abhd15-silenced 3T3-L1 cells only showed a ~30% decrease in Abhd15 mRNA expression (Figure 4B), and did not show any decrease in Abhd15 expression after 2 weeks of culturing (data not shown). Nevertheless, compared to control cells the cells with reduced Abhd15 expression showed a slower proliferation rate, reflected by a decrease in cell count by 30-40% 48 hours after seeding a defined number of cells (Figure 4C). This observation was confirmed by a colorimetric proliferation assay (MTS), revealing a reduction in proliferation of preconfluent Abhd15-silenced cells by ~20% (Figure 4D). In line with this, cells stably overexpressing Abhd15 (Panel 1 in Figure S1) showed a slightly increased cell proliferation (Panel 3 in Figure S1).

To get a better insight into the changed proliferation of Abhd15-silenced cells, their cell cycle was analyzed in more detail using BrdU FACScan. The analysis revealed an increased SubG1 peak, without any changes in the S phase in Abhd15-silenced 3T3-L1 cells (Figure 4E, Panel 4 in Figure S1). As the SubG1 peak reflects apoptotic cells, whereas the S phase shows cells in the interphase, these results indicate increased apoptosis, rather than a defect in cell division, as a cause for the reduced cell number. Further, western blot analysis of B-cell lymphoma 2 (BCL-2) and BCL-2-associated X protein (BAX), both essential regulators of apoptosis [38], revealed decreased protein levels of the pro-survival regulator BCL-2, and increased protein levels of the pro-apoptotic regulator BAX (Figure 4F, 4G). Finally, a caspase 3/7 assay, showing a more than 2-fold increase in caspase activity in Abhd15-silenced cells (Figure 4H), provided the last hint that apoptosis is increased in preconfluent Abhd15-silenced 3T3-L1 cells. In accordance with these findings, induced apoptosis (provoked by treatment of preconfluent 3T3-L1 cells with palmitic acid concentrations leading to conditions from non-apoptotic (100 µM) to highly apoptotic (500 µM) for 24 hours [39]) resulted in a massive increase of Abhd15 mRNA expression in a dose-dependent manner (Figure 4I).

Together these results demonstrate a connection of Abhd15 levels and apoptosis and suggest that a sufficient amount of Abhd15 is necessary to keep apoptotic signaling in check.

## Discussion

In this study, we provide conclusive evidence that Abhd15 is a direct and functional target gene of PPARγ and an essential factor for adipogenesis. Interestingly, while Abhd15 expression increases during adipogenesis, it decreases in the presence of high levels of FFAs, as observed in diet- [31] and genetically [32] induced obesity, fasting [33] and aging [34], as well as upon FFA treatment of cultured mature adipocytes. Furthermore, we show that knock-down of Abhd15 in preadipocytes leads to increased apoptosis, and that induced apoptosis in turn strongly increases Abhd15 expression.

Our results demonstrate that the proximal promoter of Abhd15 contains a functional PPARγ binding site. This adds Abhd15 to the large group of direct and functional PPARγ targets, of which many are important adipogenic players, such as FABP4, CD36, GLUT4, APMAP, and ARXES [15,16,40,41]. Like other adipogenic and PPARγ target genes [40], the expression of Abhd15 is strongly upregulated during adipogenic differentiation. Moreover, when cells were exposed to the PPARγ agonist rosiglitazone, Abhd15 expression was increased similarly like the above mentioned adipogenic genes [40]. 

Abhd15 is mainly expressed in murine adipose tissues and upregulated during *in vitro* adipogenesis, pointing toward a role of ABHD15 in adipocyte development. Although Chavez at al. could not detect a differentiation defect in Abhd15-silenced 3T3-L1 cells [17], we clearly show that Abhd15 expression is required for adipogenesis, as Abhd15-silenced 3T3-L1 cells were unable to increase the expression levels of adipogenic marker genes, leading to reduced lipid accumulation. The deviating result on differentiation upon Abhd15 silencing between our study and the study of Chavez et al. could be explained by increased silencing efficiency obtained with our approach. Chavez et al. reached 50% silencing on day 7 of differentiation [17], while our results are based on 80% Abhd15 silencing. As transient silencing in fully differentiated cells did not evoke any changes of the mature adipocyte phenotype, we conclude that Abhd15 lacks a role in the maintenance of the mature adipogenic status. Stable silencing of Abhd15 in 3T3-L1 cells lowers Pparγ expression levels as soon as 12 hours after induction of differentiation. Therefore, expression of adipogenic markers was not induced in Abhd15 stably silenced 3T3-L1 cells, including Abhd15 itself, leading to an improved silencing efficiency from 30% in preconfluent cells to 80% during differentiation. Searching for a cause for the differentiation defect prior to Pparγ induction, we observed that Abhd15-silenced cells proliferated slower than control cells, shown by reduced cell counts and a colorimetric proliferation assay. Cell cycle analysis revealed no change in the S phase, but an increased SubG1 peak. These observations, together with pro-death regulation of the apoptosis marker BCL-2 and BAX, and increased caspase 3/7 activity, hint to apoptosis as causal for the proliferation defect. Hence, the low silencing efficiency of only ~30% in preconfluent cells as well as the observed loss of silencing after 2 weeks of culturing could be explained by an apoptosis-mediated “dilution” of cells with high Abhd15 knockdown during prolonged culturing. The fact that reduced expression of Abhd15 led to increased apoptosis, suggests to us that Abhd15 is required for cell survival, and therefore probably has an anti-apoptotic function. On the other hand, induced apoptosis highly increased Abhd15 mRNA expression, which in itself could indicate a pro-apoptotic role. Taken together though, the apoptosis-mediated increase of Abhd15 could be seen as a compensatory (unsuccessful) attempt to reduce apoptotic signaling. Therefore, it is tempting to hypothesize that Abhd15, besides being a novel putative adipogenic player, also plays a role in the control of apoptosis, perhaps as an apoptosis-protecting factor, at least in the investigated cell type. 

Previously, it was shown that Abhd15 expression regulates PDE3B expression in 3T3-L1 cells [17]. Therefore, reduction of PDE3B could contribute to the observed phenotype of Abhd15-silenced cells. Amongst others, PDE3B is able to hydrolyze cAMP and thereby takes part in the regulation of glucose and lipid metabolism [42]. Reduced PDE3B could result in increased cAMP levels, which in turn can have pro- or anti-apoptotic effects [43]. However, these effects depend on the cell type [43].

Previous studies showed that apoptosis is increased in adipocytes of mice with diet-induced obesity [12]. These mice also have increased levels of FFAs [31], which per se are known to induce apoptosis [44–46]. However, the complete mechanism connecting these two characteristics of the diet-induced obesity phenotype is still elusive. Our data suggest that Abhd15 could be a key player in this context, as we found Abhd15 expression to be consistently decreased *in vivo* and *in vitro* upon conditions of elevated FFA levels. In adipocytes, superfluous FFAs can activate a number of different serine kinases, leading to inhibition of insulin signaling [47] and, in turn, to reduced Akt activation. Akt signaling has been shown to phosphorylate Abhd15 [17,18]. As a result, high levels of FFAs might not only lead to decreased mRNA expression of Abhd15, but also influence the phosphorylation state of the remaining protein. For these reasons it is tempting to speculate that reduction/impairment of “protecting Abhd15” by increased FFA content leads to induced apoptosis and its further consequences, like recruitment of adipose tissue macrophages, insulin resistance, and development of fatty liver [12].

In conclusion, our results show that Abhd15 is a functional PPARγ target gene and required for adipogenesis. In addition, we provide evidence that Abhd15 expression levels are tightly connected to apoptosis. While decreased expression of Abhd15 evokes apoptosis, a striking increase of Abhd15 expression can be found upon induction of apoptosis, proposing Abhd15 as a protective factor against apoptosis. Together with its intricate regulation by FFAs, Abhd15 might be an intriguing new target in obesity and diabetes research, as it impacts on adipogenesis and apoptosis, both factors crucially determining adipose cell number and size. 

## Supporting Information

Figure S1 **3T3-L1 cells were infected with lentiviral particles obtained from phoenix cells transfected with either empty pMSCVpuro vector (pMSCVpuro) or a vector containing the Abhd15 gene (pMSCV-Abhd15).** After transduction, 3T3-L1 cells were selected with puromycin and expanded as a mixed population. **1**. Relative mRNA expression of Abhd15 in preconfluent 3T3-L1 cells stably overexpressing Abhd15 compared to control cells. **2**. Overexpression of Abhd15 does not affect adipogenesis when compared to control cells, indicated by similar neutral lipid staining on day 7 of differentiation. **3**. Cell proliferation was slightly increased in Abhd15 overexpressing preconfluent 3T3-L1 cells, shown by an upwards trend in the cell number 48 hours after seeding. **4**. 3T3-L1 cells were infected with lentiviral particles coding for Abhd15 shRNA (Abhd15_sil) using a non-target shRNA as control (ntc), selected for puromycin resistance and expanded as a mixed population. Analysis of the certain stages of cell division, using BrdU FACScan, revealed no differences in the S phase peak between preconfluent Abhd15-silenced 3T3-L1 and control cells. Data is presented as mean ± SD from at least three independent experiments.
(TIF)
