# Supplementary figures and images for: α/β-Hydrolase Domain Containing Protein 15 (ABHD15) – an Adipogenic Protein Protecting from Apoptosis
Source: PLoS One. 2013 Nov 13;8(11):e79134. doi: 10.1371/journal.pone.0079134 (PMC3827343; doi:10.1371/journal.pone.0079134)

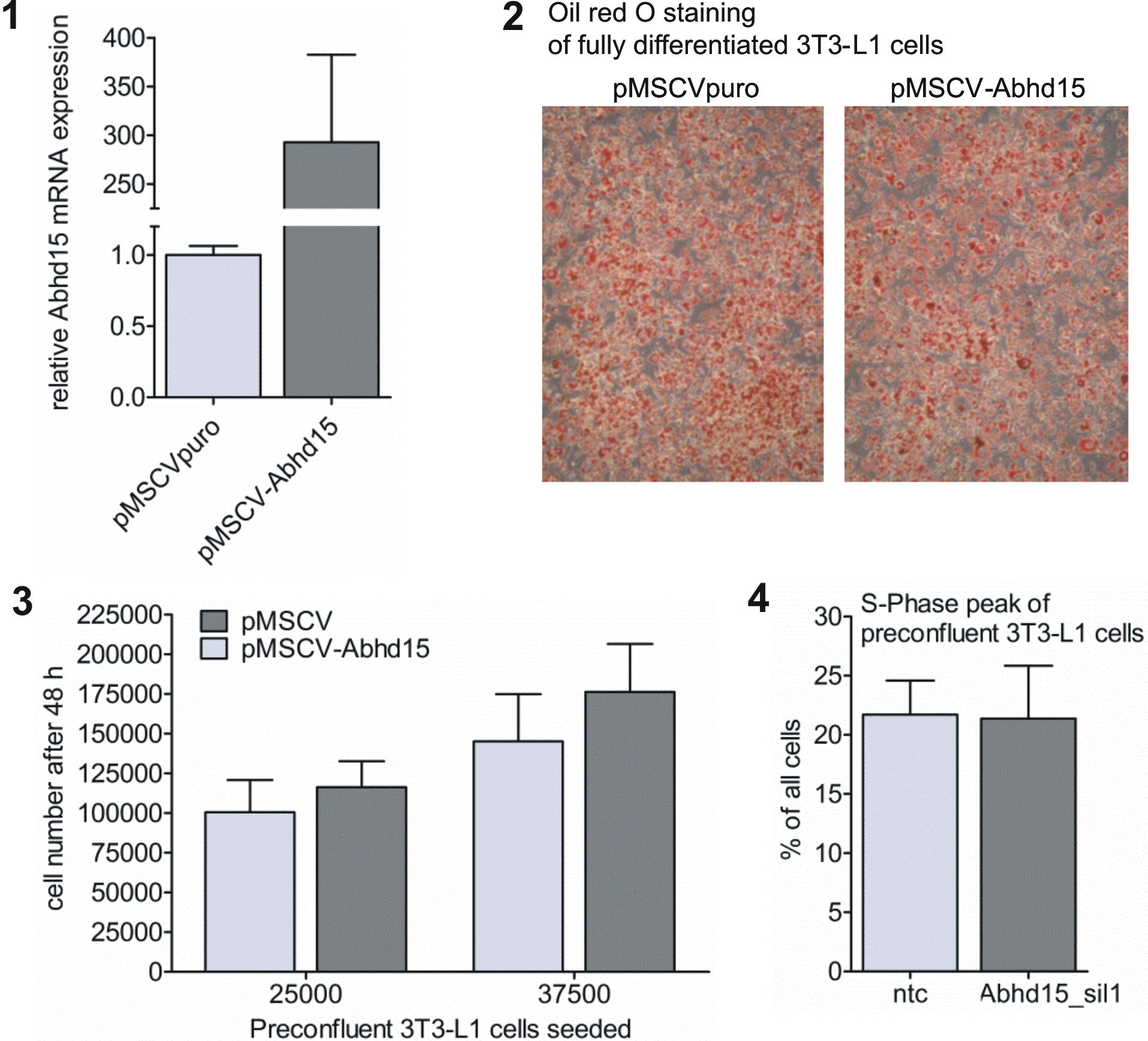

Supplement: Figure S1 — 3T3-L1 cells were infected with lentiviral particles obtained from phoenix cells transfected with either empty pMSCVpuro vector (pMSCVpuro) or a vector containing the Abhd15 gene (pMSCV-Abhd15). After transduction, 3T3-L1 cells were selected with puromycin and expanded as a mixed population. 1. Relative mRNA expression of Abhd15 in preconfluent 3T3-L1 cells stably overexpressing Abhd15 compared to control cells. 2. Overexpression of Abhd15 does not affect adipogenesis when compared to control cells, indicated by similar neutral lipid staining on day 7 of differentiation. 3. Cell proliferation was slightly increased in Abhd15 overexpressing preconfluent 3T3-L1 cells, shown by an upwards trend in the cell number 48 hours after seeding. 4. 3T3-L1 cells were infected with lentiviral particles coding for Abhd15 shRNA (Abhd15_sil) using a non-target shRNA as control (ntc), selected for puromycin resistance and expanded as a mixed population. Analysis of the certain stages of cell division, using BrdU FACScan, revealed no differences in the S phase peak between preconfluent Abhd15-silenced 3T3-L1 and control cells. Data is presented as mean ± SD from at least three independent experiments. (TIF) [file pone.0079134.s001.tif]
